# Pyroptosis: A promising therapeutic target for noninfectious diseases

**DOI:** 10.1111/cpr.13137

**Published:** 2021-09-29

**Authors:** Tong Li, Guangjuan Zheng, Ben Li, Lipeng Tang

**Affiliations:** ^1^ State Key Laboratory of Dampness Syndrome of Chinese Medicine the Second Affiliated Hospital of Guangzhou University of Chinese Medicine Guangzhou China; ^2^ Department of Pharmacology of Traditional Chinese Medicine The Second Clinical College of Guangzhou University of Chinese Medicine Guangzhou China; ^3^ Department of Pharmacy The Second Clinical College of Guangzhou University of Chinese Medicine Guangzhou China; ^4^ Department of Pathology The Second Clinical College of Guangzhou University of Chinese Medicine Guangzhou China

## Abstract

Pyroptosis, which is characterized by gasdermin family protein‐mediated pore formation, cellular lysis and the release of pro‐inflammatory cytokines, is a form of programmed cell death associated with intracellular pathogens‐induced infection. However, emerging evidence indicates that pyroptosis also contributes to sterile inflammation. In this review, we will first illustrate the biological process of pyroptosis. Then, we will focus on the pathogenic effects of pyroptosis on multiple noninfectious disorders. At last, we will characterize several specific pyroptotic inhibitors targeting the pyroptotic signalling pathway. These data demonstrate that pyroptosis plays a prominent role in sterile diseases, thereby providing a promising approach to the treatment of noninfective inflammatory disorders.

## Graphical abstract

Pyroptosis contributes to multiple noninfectious disorders including neuronal diseases, sterile inflammatory diseases, cancer, atherosclerosis, acute injury, adverse pregnancy complications and autoimmune diseases via lytic cell death, cytokines releases, dysfunction of organelles and auto‐antigens release. Meanwhile, pyroptosis can also inhibit the development of some specific tumor via pyroptotic cell death and anti‐tumor immunity. Red line and text mean promotion. Green line and text indicate suppression. AT1‐AA, angiotensin II type 1 receptor autoantibody.

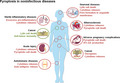

## 1 INTRODUCTION

Pyroptosis, a burgeoning form of programmed cell death, is characterized by gasdermin‐mediated pore formation in the plasma membrane.^1^ It plays a critical role in innate immunity against bacterial and viral infections.^2^ However, pyroptosis might be a double‐edged sword. On the one hand, excessive pyroptotic cell death might have detrimental effects on the host responses. In agreement, various studies indicate that excessive pyroptosis results in endotoxic sepsis shock.^3^, ^4^ On the other hand, pyroptosis is also involved in multiple noninfectious diseases. Emerging evidence has recently demonstrated that pyroptosis contributes to the pathology of multiple noninfectious disorders, including sterile inflammatory diseases, autoimmune disorders, nervous system diseases, tumours, atherosclerosis, acute injuries and adverse pregnancy complications.^5^, ^6^, ^7^, ^8^ As pyroptosis likely plays a contributory role in these noninfectious diseases, the identification of small molecular inhibitors targeting pyroptotic signalling pathway is of great interest. In this review, we will address the pathogenic role of pyroptosis in several non‐infective inflammatory diseases. Moreover, we will highlight the current therapeutic strategies that target pyroptotic proteins and signalling pathways.

## 2 PYROPTOSIS

Pyroptosis, which was originally described in 2000,^9^ is a form of lytic and inflammatory cell death characterized by pore formation in the plasma membrane, swelling and rupture of cell and cytosolic contents leakages.^10^ Therefore, in 2001, Cookson and Brennan defined this kind of inflammatory program cell death as pyroptosis (‘pyro’ means ‘fire or fever’ and ‘ptosis’ relates to ‘a falling’ in Greek).^11^ Emerging evidence indicates that pyroptosis takes an important role in host defences against intracellular pathogens infection and various noninfectious diseases,^12^, ^13^ however, the cellular activation modes of pyroptosis remained unclear until recently.

### 2.1 Pro‐inflammatory caspase‐mediated pyroptosis

Inflammasomes are multi‐protein complexes that assemble in the cytosol after sensing pathogen‐associated molecular patterns (PAMPs) or danger‐associated molecular patterns (DAMPs).^14^ Inflammasomes can be divided into canonical inflammasomes and noncanonical inflammasomes according to their different components. Canonical inflammasomes are composed of a sensor protein [including AIM2 (Absent in melanoma 2), CARD8 (caspase recruitment domain‐containing protein 8), NLRP1 (nucleotide‐binding domain and leucine‐rich repeat pyrin‐domain containing protein 1), NLRP3 (nucleotide‐binding oligomerization domain, leucine‐rich repeat and pyrin domain‐containing 3), NLRC4 (nucleotide‐binding domain leucine‐rich repeat‐containing protein family caspase activation and recruitment domain‐containing protein 4) and pyrin], an adaptor apoptosis‐associated speck‐like (ASC) and caspases‐1, whereas noncanonical inflammasomes are just assembled by human caspase‐4/5 (mouse orthologs caspase‐11).^15^, ^16^ The canonical and noncanonical inflammasomes can be activated by a repertoire of infectious and sterile stimuli. For an instant, NLRP1 inflammasome responds to Bacillus anthracis. NLRC4 inflammasome senses Salmonella. NLRP3 responds to ROS and K^+^. AIM2 inflammasome detects DNA virus. Pyrin inflammasome discerns toxins. In addition, noncanonical inflammasome senses lipopolysaccharide (LPS) of Gram‐negative Bacilli.^17^

**FIGURE 1:**
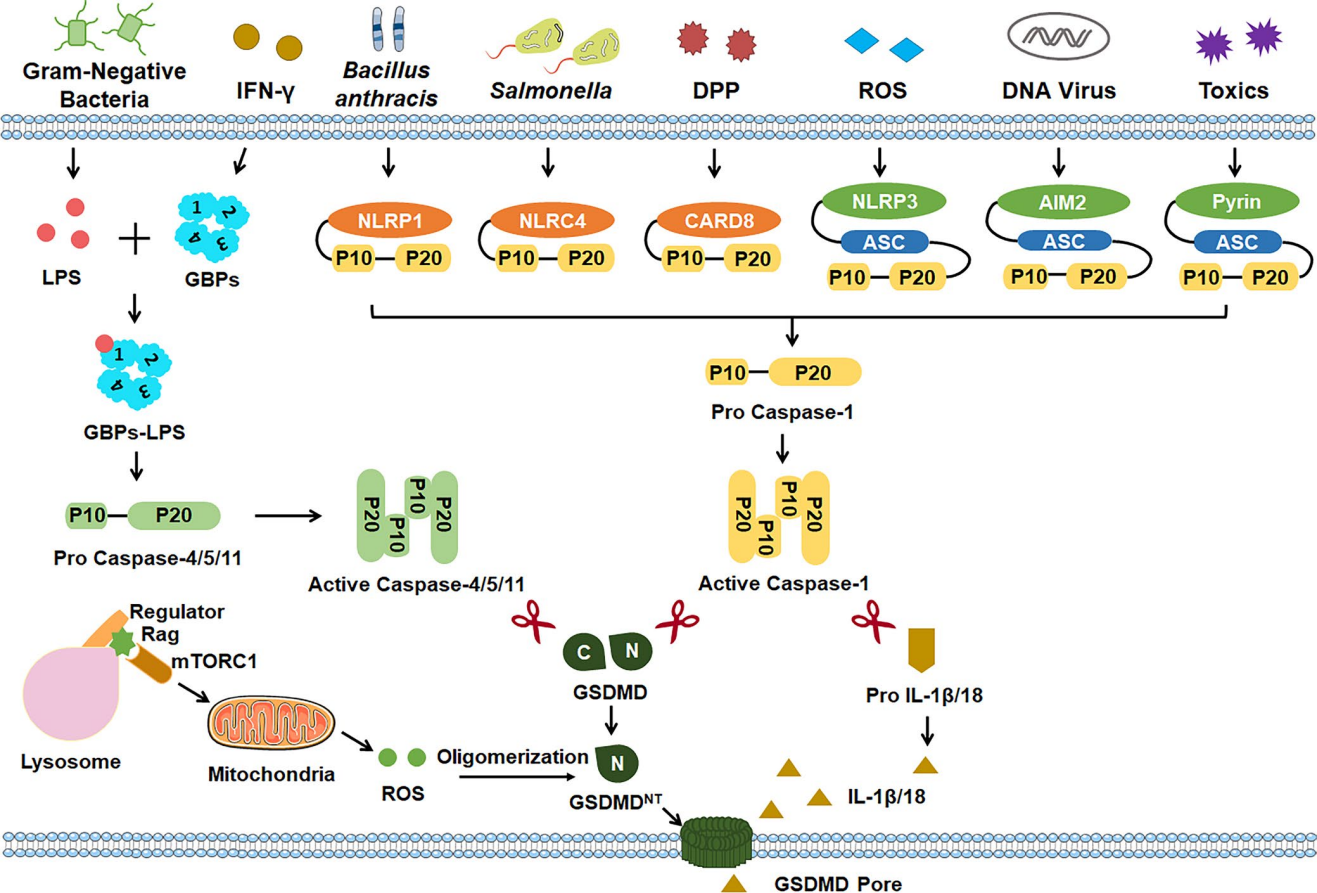
Pro‐inflammatory caspases‐triggered pyroptosis. Gasdermin D (GSDMD)‐mediated pyroptosis can be triggered by pro‐inflammatory caspases downstream of canonical or noncanonical inflammasomes. The canonical inflammasome sensors, including NLR family pyrin domain‐containing 1 (NLRP1), NLR family CARD domain containing 4 (NLRC4), caspase recruitment domain‐containing protein 8 (CARD8), NOD‐like receptor protein 3 (NLRP3), absent in melanoma 2 (AIM2) and pyrin, detect diverse stimuli and activate caspase‐1 to cleave GSDMD. IFN‐γ or cytosolic LPS responder guanylate‐binding proteins (GBPs), which are key regulators for noncanonical inflammasome activation, serve as a platform for caspase‐4,5 (or caspase‐11 in mice) recruitment and subsequently cleave GSDMD. Once cleaved, GSDMD^NT^ fragment can insert into the lipid bilayer of the plasma membrane and subsequently oligomerize to form ring‐like membrane‐spanning pores by regulator‐rag‐mTORC1‐mitochondrial reactive oxygen species (ROS) pathway. ASC, apoptosis‐associated speck‐like protein containing a CARD; DPP, dipeptidyl‐peptidase; GSDMD^NT^, the N‐terminal of GSDMD protein; IL‐1β, interleukin‐1β; LPS, lipopolysaccharide; mTORC1, the mechanistic target of rapamycin complex 1

Of interest, pyroptosis is first identified to be triggered by caspase‐1 downstream of canonical inflammasomes (Figure 1). In 2015, He et al and Shi et al^18^, ^19^ indicated that caspase‐1, which is activated downstream of NLRP3, pyrin and AIM2 inflammasomes activation, can induce pyroptotic cell death by cleaving gasdermin D (GSDMD) into a 31 kDa pore‐forming N‐terminal GSDMD^NT^ fragment in marrow‐derived macrophages (BMDMs). Similarly, Linder et al^20^ showed that caspase‐1, which is activated downstream of CARD8 inflammasome upon dipeptidyl‐peptidase (DPP) inhibition, induces pyroptosis in human resting T cells. Mechanistically, the GSDMD^NT^ fragment, which is cleaved by active caspase‐1, binds to and then inserts into the lipid bilayer of the plasma membrane.^21^, ^22^ Using high‐resolution atomic force microscopy (AFM) and cryo‐electron microscopy (cryo‐EM), Mulvihill et al and Xia et al^23^, ^24^ further showed that the β1–β2 loop of GSDMD^NT^ prepore is critical for its insertion into the lipid bilayer. They found that the hydrophobic tips of β1–β2 loop serve as an anchor for insertion whilst the surrounding basic residues interact with the acidic lipids. Once inserted, GSDMD^NT^ fragment oligomerizes and forms ring‐like membrane‐spanning pores through regulator‐rag‐mTORC1‐mitochondrial reactive oxygen species (ROS) pathway.^25^ Interestingly, Xia et al^24^ proposed that GSDMD^NT^ prepore, which enriches with negative potentials, preferentially releases positively charged mature IL‐1β but not negatively charged pro‐IL‐1β through an electrostatics‐dependent way.

Additionally, pyroptosis can also be caused by caspase‐4/5 (human) or caspase‐11 (mouse) downstream of the non‐canonical inflammasome (Figure 1). In 2015, Kayagaki et al, Shi et al and He et al^18^, ^19^, ^26^ simultaneously demonstrated that caspase‐4/5 or caspase‐11, which is activated by LPS of Gram‐negative Bacilli, can also cause GSDMD cleavage and subsequent GSDMD‐mediated pyroptosis in macrophages. Intriguingly, Santos et al recently demonstrated that interferon‐induced guanylate‐binding proteins (GBPs) function as an important cytosolic regulator for LPS‐induced noncanonical inflammasome activation and pyroptosis. Mechanistically, GBPs serve as a bonafide cytosolic LPS sensor and assemble a platform for caspase‐4 recruitment.^27^

### 2.2 Pro‐apoptotic caspase‐induced pyroptosis

**FIGURE 2:**
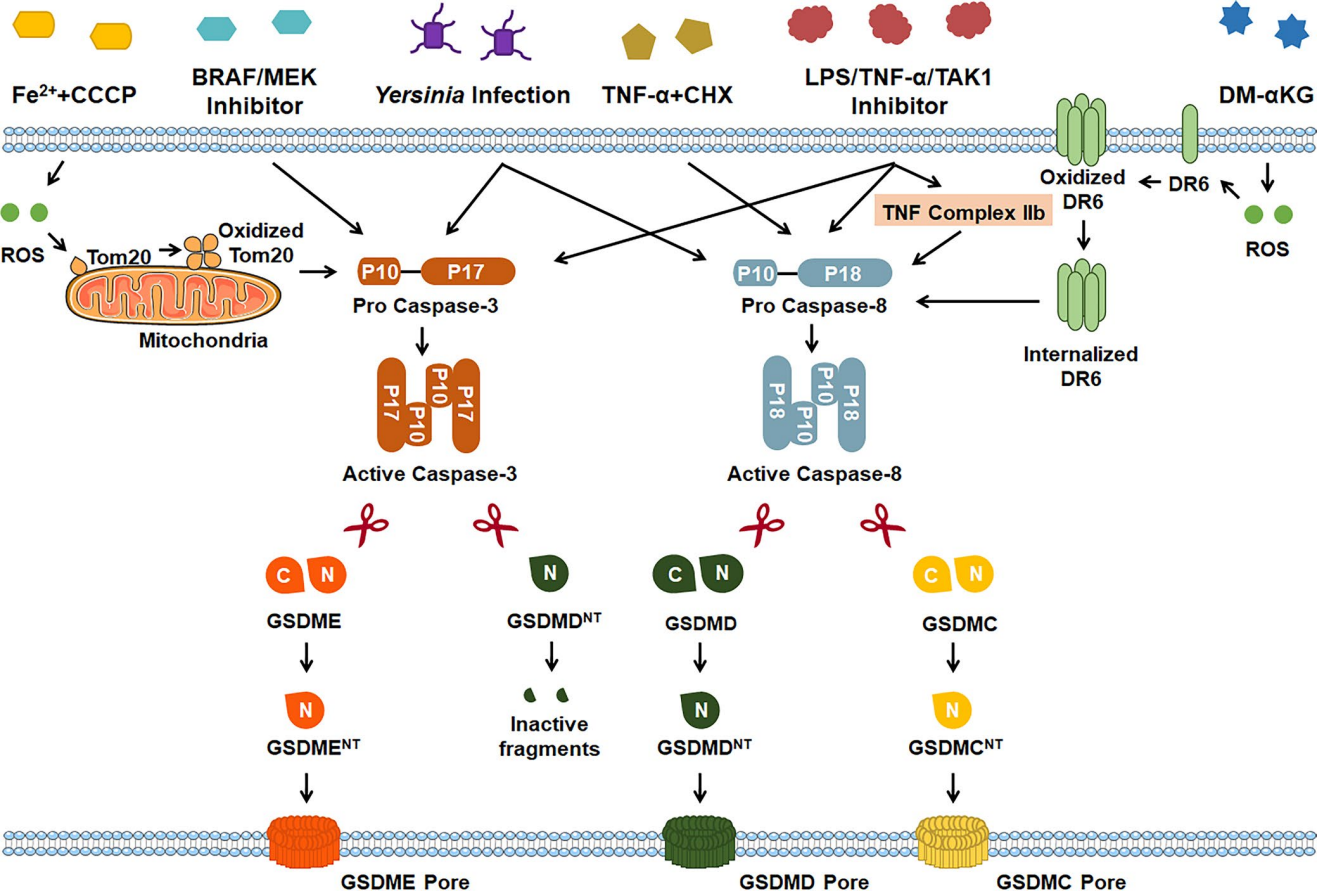
Pro‐apoptotic caspases‐mediated pyroptosis. Gasdermin E (GSDME)‐, GSDMD‐ or Gasdermin C (GSDMC)‐dependent pyroptosis can be caused via pro‐apoptotic caspases after multiple stimuli, including Fe^2+^ and carbonyl cyanide 3‐chlorophenylhydrazone (CCCP) co‐treatment, kinase B‐raf (BRAF) inhibitors and MAPK/ERK kinase (MEK) inhibitors co‐treatment, TNF‐α treatment, LPS/TNF‐α/TGF‐β‐activated kinase 1 (TAK1) inhibitor co‐stimulation, *Yersinia* infection and DM‐α‐KG. CCCP, Carbonyl cyanide 3‐chlorophenylhydrazone; CHX, cycloheximide; DM‐α‐KG, dimethyl‐α‐ketoglutarate; DR6, death receptor 6; GSDMC^NT^, the N‐terminal of GSDMC protein; GSDMD^NT^, the N‐terminal of GSDMD protein; GSDME^NT^, the N‐terminal of GSDME protein; ROS, reactive oxygen species; TGF‐β, transforming growth factor‐β

Apart from pro‐inflammatory caspases, several studies have demonstrated that pyroptosis can be induced via pro‐apoptotic caspases independent of the canonical or noncanonical inflammasomes (Figure 2).

Caspase‐8, which is a classical pro‐apoptotic caspase, can lead to pyroptosis downstream of a broad array of different stimuli. Sarhan et al, Chen et al and Orning et al^28^, ^29^, ^30^ demonstrated that caspase‐8 cleaves GSDMD into GDSMD^NT^ fragments and consequently drives pyroptosis upon *Yersinia* infection or LPS/TNF‐α/TAK1 inhibitor co‐stimulation in cultured BMDMs and in mice. Mechanistically, TNF‐α/TAK1 inhibitor, which can promote TNF complex IIb formation, induces the autoprocessing of caspase‐8 into active p18 fragment and forms dimerization, finally triggering GSDMD cleavage and GDSMD^NT^‐mediated pyroptosis.^31^ Interestingly, caspase‐8 can also shear gasdermin C (GSDMC) into GDSMC^NT^ fragments and subsequently switch apoptosis to pyroptosis in breast cancer cells with TNF‐α treatment.^32^ Consistently, Zhang et al^33^ recently showed that the caspase‐8, which is recruited to ROS‐oxidized and internalized death receptor 6 (DR6) after application with dimethyl‐α‐ketoglutarate (DM‐α‐KG), can cleave GSDMC at Asp240 and subsequently trigger GSDMC‐mediated pyroptotic cell death in many human gastric cancer cells (SGC‐7901 and BGC‐823), human colon cancer cells (HCT116) and human hepatoma cells (Huh7).

In contrast to caspase‐8, caspase‐3 plays a much more complex role in pyroptosis. Caspase‐3 can cleave gasdermins E (GSDME) and consequently trigger GSDME‐dependent pyroptosis downstream of LPS/TNF‐α/TAK1 inhibitor co‐stimulation or *Yersinia* infection or BRAF/MEK inhibitor co‐treatment.^28^, ^34^ Furthermore, Zhou et al demonstrated that caspase‐3‐mediated GSDME cleavage is involved in pyroptosis induced by iron‐activated ROS.^35^ However, caspase‐3 can counteract the GSDMD‐dependent pyroptosis by further cleaving the pyroptotic‐GSDMD^NT^ fragment into a small inactive fragment after TNF‐α and TAK1i co‐stimulation.^28^, ^29^, ^31^ Future work should further explore the precise balance and crosstalk between caspase‐8‐promoted and caspase‐3‐suppressed GSDMD‐mediated pyroptosis.

### 2.3 Granzyme‐triggered pyroptosis

**FIGURE 3:**
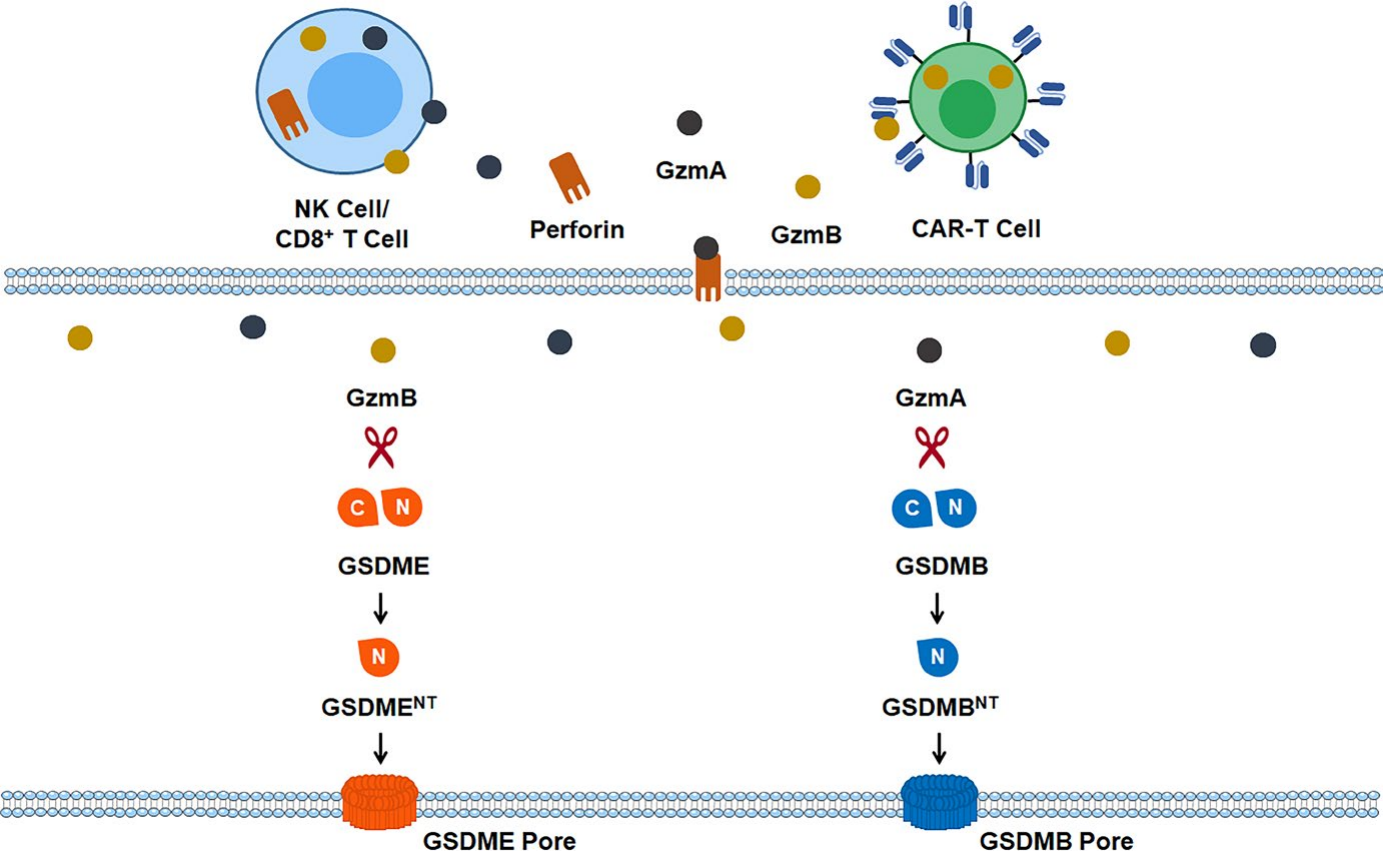
Granzyme‐induced pyroptosis. GSDMB‐ or GSDME‐regulated pyroptosis can be induced through granzymes, which are released from killer cytotoxic lymphocytes (including NK cell/ CD8^+^ T cell) and chimeric antigen receptor T cell (CAR‐T cell). CAR‐T cell, chimeric antigen receptor T cell; GzmA, granzyme A; GzmB, granzyme B; NK cells, natural killer cells

In 2020, two independent studies illustrated for the first time that pyroptosis can be triggered through granzyme, which is independent of inflammatory or pro‐apoptotic caspases (Figure 3). Zhang et al^36^ found that serine protease Granzyme B (GzmB) from cytotoxic lymphocytes can induce GSDME cleavage directly in target cells. Similarly, Zhou and colleagues gave another evidence that Granzyme A (GzmA), which is also released from cytotoxic lymphocytes, can cleave gasdermins B (GSDMB) directly and eventually give rise to GSDMB‐mediated pyroptosis in target cells.^37^ These studies rewrite the conclusion that pyroptosis can only be activated by caspases, which expands our understanding of the activation modes of pyroptosis.

## 3 PYROPTOSIS IN NONINFECTIOUS DISEASES

**FIGURE 4:**
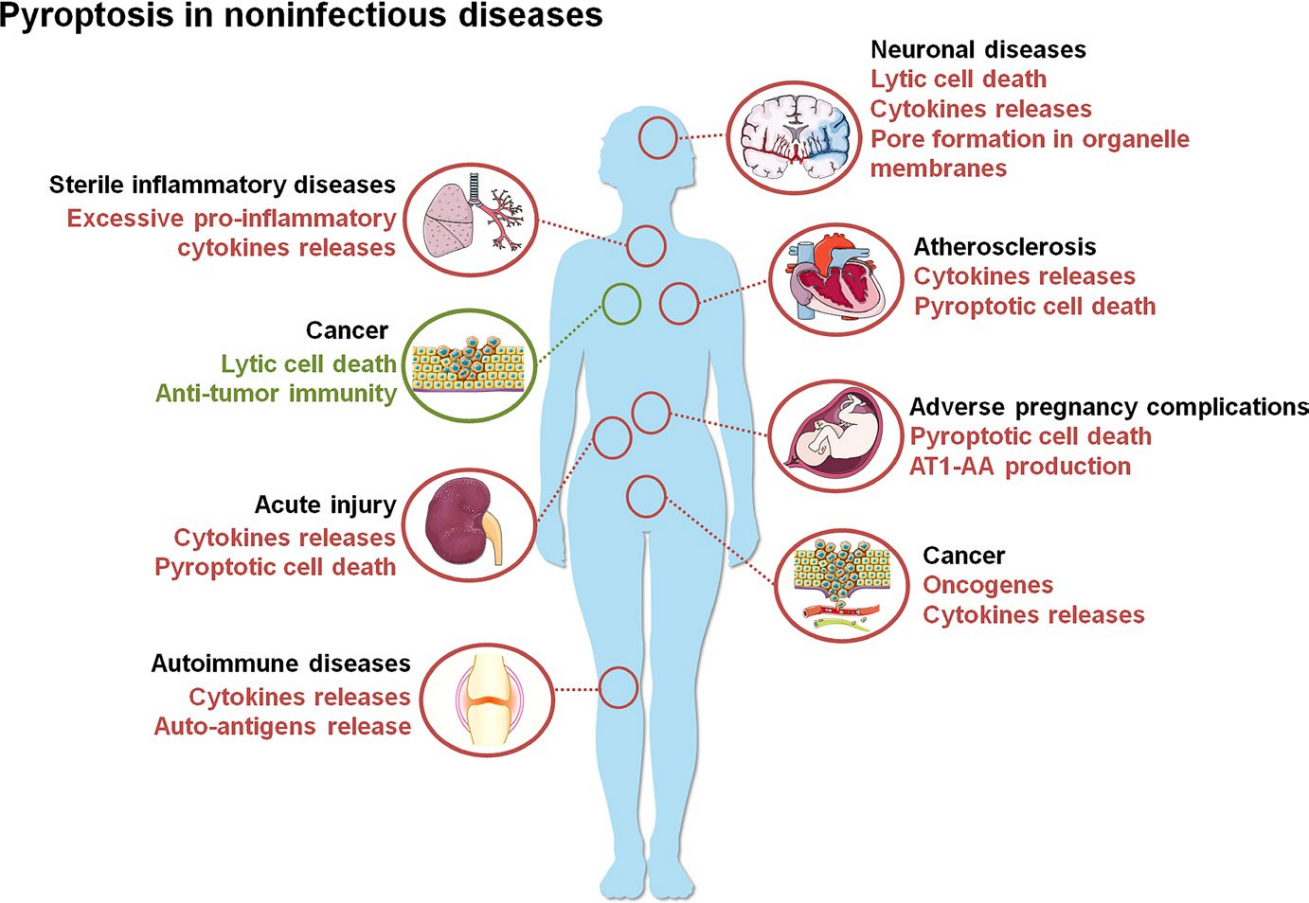
Pyroptosis in noninfectious diseases. Pyroptosis contributes to multiple noninfectious disorders including neuronal diseases, sterile inflammatory diseases, cancer, atherosclerosis, acute injury, adverse pregnancy complications and autoimmune diseases via lytic cell death, cytokines releases, dysfunction of organelles and auto‐antigens release. Meanwhile, pyroptosis can also inhibit the development of some specific tumours via pyroptotic cell death and anti‐tumour immunity. Redline and text mean promotion. Greenline and text indicate suppression. AT1‐AA, angiotensin II type 1 receptor autoantibody

In normal physiology, pyroptosis plays a critical role in anti‐microbial innate immune defences.^38^, ^39^ However, excessive cell deaths and inflammatory responses caused by pyroptosis may also have deleterious effects on various sterile, noninfectious disorders (Figure 4).

### 3.1 Pyroptosis in sterile inflammatory diseases

The sterile inflammatory response, which is in the absence of infection, is required for organ development, tissue repair and host defence. However, dysregulated sterile inflammation may lead to many inflammatory diseases, including lung inflammation, type 2 diabetes and liver sterile diseases. Given the critical effects of pyroptosis on driving inflammation, it has been hypothesized that pyroptosis may function as a potential contributor in several sterile inflammatory diseases.

Asthma is a chronic inflammatory disease of the airways with the common clinical features of recurring wheezing, chest tightness and breathlessness. Although both genetic and environmental factors contribute to asthma, the pathogenesis of asthma is still ill‐defined. Previously, a series of genome‐wide association studies (GWAS) indicated that GSDMA and GSDMB polymorphisms are associated with asthma.^40^, ^41^, ^42^ However, the mechanism whereby GSDMA and GSDMB promote the onset of asthma is largely unknown. Recently, Panganiban et al^43^ proved that GSDMB might contribute to asthma through GSDMB‐dependent pyroptosis in airway epithelial cells. Additionally, a splicing variant (rs11078928) of GSDMB can reduce asthma risk as this variant can abolish GSDMB‐mediated pyroptosis by deleting 13 amino acids in the N‐terminus of GSDMB. Furthermore, inflammasomes (such as NLRP3) and caspases (such as caspase‐1/11) have been reported to participate in asthma. Toluene diisocyanate (TDI) was found to exacerbate asthmatic airway inflammation by inducing NLRP3 inflammasome activation. Mechanistically, active NLRP3 inflammasome in epithelial cells activate caspase‐1 to cleave GSDMD, finally increasing the IL‐1β release and aggravating the airway inflammation in asthma.^44^ Moreover, Zaslona et al^45^ identified that caspase‐11‐driven pyroptosis in macrophages is a critical regulator of allergic airway inflammation. Taken together, these studies advance our knowledge of the contributory role of pyroptosis in asthma.

Type 2 diabetes (T2D) is a chronic disease characterized by hyperglycemia and relative insulin deficiency due to the progressive loss of insulin secretion. Islet inflammation has proved to be a major pathological cause of insulin secretion deficiency. Recently, Chang et al^46^ identified that NLRP3 inflammasome‐induced pyroptosis contributes to islet inflammation in type 2 diabetes mellitus patients and rats. Mechanistically, NEK7 (NIMA‐related kinase 7), which can be suppressed by miR‐23a‐3p, is highly expressed in type 2 diabetes mellitus patients and rats. Upregulated NEK7 triggers NLRP3 inflammasome activation and then promotes caspase‐1‐GSDMD‐mediated pyroptotic cell death and IL‐1β releases in BMDM, eventually resulting in islet inflammation and T2D onset. Moreover, pyroptosis also exerts contributory effects on multiple diabetic complications, including diabetic cardiomyopathy and diabetic retinopathy. Mechanistically, ROS‐activated NLRP3 or AIM2 inflammasome leads to pyroptotic cell death in diabetic cardiomyoblasts via the GSDMD pathway.^47^, ^48^ Additionally, diabetic retinal pericytes loss is partly mediated by NLRP3‐caspase‐1‐GSDMD‐triggered pyroptosis.^49^ These findings suggest that pyroptosis might be a promising therapeutic intervention for T2D.

Pyroptosis also contributes to sterile inflammatory liver diseases, including alcoholic hepatitis (AH), nonalcoholic fatty liver disease (NAFLD) and heatstroke‐induced liver injury. For example, Khanova et al^50^ found that pyroptosis induced by the caspase11/4‐GSDMD pathway is implicated in the pathogenesis of AH. Mechanistically, activated caspase‐11/4 cleaves GSDMD and consequently induces GSDMD‐mediated pyroptosis in hepatic macrophages and hepatocytes, ultimately aggravating hepatocyte death and hepatic bacterial load. In addition, Xu et al^51^ showed that GSDMD‐mediated pyroptosis plays a key role in the pathogenesis of NAFLD by promoting hepatic nutritional fibrosis, lipogenesis and extensive inflammatory responses in cultured hepatocytes and in methionine‐choline deficient (MCD)/ high‐fat diet (HFD)‐induced steatohepatitis mice model. Moreover, Gaul et al^52^ indicated that NLRP3 inflammasome oligomers, which are released from the pyroptotic hepatocytes, further contribute to NAFLD by inducing nearby hepatic stellate cell activation and ultimately perpetuating liver inflammation and fibrosis. Of interest, Geng et al^53^ showed that pyroptosis is also involved in heatstroke‐induced liver injury. Mechanistically, heat stress‐activated NLRP3 inflammasome triggers hepatocyte pyroptosis and consequently results in heatstroke‐induced liver injury. In summary, these studies provide experimental evidence to understand how the pyroptotic signalling pathway enhances sterile inflammation in various liver diseases, which provide potential therapeutic targets for sterile inflammatory liver diseases.

### 3.2 Pyroptosis in autoimmune diseases

Autoimmune diseases are characterized by the production of autoreactive antibodies that react with immune effector cells or host tissues. Accumulating evidence suggests that pyroptosis is involved in the pathogenesis of autoimmune diseases.

Systemic lupus erythematosus (SLE), which is an autoimmune disease with multi‐system damage, is characterized by the presence of autoreactive antibodies, immune complex formation and deposition in the, joints, kidneys and serosal membranes. Accumulated evidence indicates that pyroptosis is crucial for SLE. Pyroptosis in monocytes and macrophages, which is activated by canonical inflammasomes downstream of interaction with dsDNA/dsDNA antibody or U1 small riboprotein (U1‐snRNP)/anti‐U1‐snRNP antibody, can potentiate the inflammatory responses in SLE patients by releasing IL‐1β, IL‐18 and HMGB1.^54^, ^55^ Interestingly, recent evidence illustrated that the intact nuclei, which is released from pyroptotic monocytes or macrophages, might serve as a newly identified autoantigen for SLE.^56^ Consequently, targeting pyroptosis might be a good way to treat SLE.

Sjogren's syndrome (SS) is a chronic inflammatory autoimmune disease characterized by decreased production of saliva and tears. Lymphocytes and plasma cells‐mediated progressive destruction of the salivary and lacrimal glands plays an important role in SS. However, the mechanisms by which lymphocytes and plasma cells destruct the salivary and lacrimal glands are largely unknown. Researchers found that the pyroptotic proteins and cytokines, such as NLRP3, caspase‐1, IL‐1β and IL‐18, are significantly unregulated in SS patients.^57^, ^58^ Recently, Vakrakou et al^59^ demonstrated that accumulations of high cell‐free DNA (cf‐DNA) in the sera of SS patients systematically activate NLRP3 inflammasome and consequently result in pyroptotic cell death of infiltrating macrophages in the salivary glands, finally triggering the pathological process of Sjogren's syndrome. Additionally, pyroptosis in salivary gland epithelial cells (SGECs) also plays a critical role in SS. Park et al^60^ showed that type I interferon accelerates AIM2/NLRP3 inflammasome activation in SGECs, which ultimately contributes to SS by promoting caspase‐1‐ GSDMD‐associated pyroptosis in SGECs. Taken together, these results illustrate the importance of pyroptosis in Sjogren's syndrome; thereby, provide new targets for Sjogren's syndrome.

Rheumatoid arthritis (RA) is a disabling autoimmune disease characterized by inflammation and destruction of joints. Recently, two independent studies showed the opposite role of pyroptosis in RA: In one study, GSMDM‐dependent pyroptosis in monocytes exacerbates RA,^61^ while in another study, the pyroptosis deficiency in MRE11A^low^ T cells promotes synovial tissue injury and eventually promotes RA.^62^ These contrary discoveries suggest that cell‐type‐specific pyroptosis may have distinguishing effects on RA. Given that the joint is a tissue with complex and multiple cell types, future studies are required to further explore the exact role of monocytes/T cell/chondrocyte‐related pyroptosis in RA.

### 3.3 Pyroptosis in neuronal diseases

Accumulating evidence suggests that pyroptosis might participate in the pathology of neuronal diseases through multiple pathways. First, pyroptosis can induce perforation in the plasma membrane of neurons, microglia and astrocytes, which leads to pyroptotic cell death directly. Second, pyroptosis potentiates neuroinflammation via pro‐inflammatory cytokines release. Third, pyroptosis might cause organelle dysfunctions by forming pores in their membrane.

Ischemic stroke, the second leading cause of death in the world, is originally from blocks or plugs in a blood vessel in the brain by a blood clot. Clinical and basic studies recently indicated that neuron or microglia pyroptosis is involved in ischemic stroke. Yan et al^63^ documented that pyroptosis in neurons contributes to early ischemic neuronal injury via the Sirt1 (Sirtuin‐1)‐ROS‐TRAF6 (TNF receptor‐associated factor 6) signalling pathway. Sun et al^64^ showed that downregulated low‐density lipoprotein receptor (LDLR) promotes NLRP3‐mediated neuronal pyroptosis, ultimately leading to neuronal injury in ischemia. Additionally, GSDMD‐mediated pyroptosis in microglia, astrocytes and infiltrating macrophages downstream of canonical/non‐canonical inflammasomes activation facilitate the passage of intracellular inflammatory factors, ultimately promoting ischemic brain injury.^65^, ^66^, ^67^ Furthermore, pyroptosis in neurons might also induce mitochondria dysfunctions, finally resulting in increasing ROS levels and aggravating ischemic injuries.^63^ Combined, these studies suggest that pyroptosis might be a promising therapeutic target for ischemic stroke.

Epilepsy, a central nervous system (neurological) disorder, is characterized by recurrent episodes of paroxysmal brain dysfunction and severe neuronal loss in the hippocampus. Tan et al^68^ demonstrated that NLRP1/caspase‐1 signalling is implicated in epileptic degeneration in temporal lobe epilepsy (TLE) patients and the animal model of TLE. Similarly, Toscano et al^69^ showed that NLRP1/3 inflammasome, which is upregulated in the hippocampi of patients with TLE, contributes to TLE by upregulating the expression of caspase‐1 and IL‐β in sclerotic hippocampi. These studies were important experimental evidence that the pyroptotic pathway is involved in epilepsy, suggesting a novel strategy for epilepsy therapy.

Parkinson's disease (PD) is a progressive nervous system disorder characterized by the loss of midbrain dopamine neurons (mDAs) in the substantia nigra pars compacta (SNpc). Emerging studies have revealed the contributory roles of pyroptosis in PD. Zeng et al^70^ showed that 1‐methyl‐4‐phenylpyridinium (MPP^+^) can trigger GSDMD‐mediated pyroptosis by NLRP3/caspase‐1 pathway in an *in vitro* PD model, suggesting that pyroptosis acts as an inflammatory contributor in PD progression. Endogenous miR‐135b, which is downregulated in PD, suppresses MPP^+^‐induced pyroptosis through inhibiting FOXO1 (Forkhead Box Protein O1) /TXINP (Thioredoxin Interacting Protein)/ NLRP3/caspase‐1/GSDMD/pyroptosis axis. Additionally, lncRNA HOTAIR, which is unregulated in MPP^+^‐induced SH‐SY5Y cells and PD mice, promotes NLRP3‐mediated pyroptotic neuronal damage through mediating miR‐326/ELAVL1 (ELAV‐like Protein 1) pathway.^71^ These studies provide potential therapeutic targets for PD from the perspective of pyroptosis.

Alzheimer's disease (AD) is a common neurodegenerative disease characterized by cognitive decline and dementia. β‐amyloid (Aβ)‐induced senile plaque (SP) in the extracellular space, abnormally phosphorylated tau protein aggregation‐mediated neurofibrillary tangles (NFT), neurons death and vascular amyloidosis in the brain are the main pathological features of AD. Recently, Shen et al^72^ showed that GSDMD, which is the major executor protein of pyroptosis may act as a diagnostic biomarker for AD patients. Additionally, the pyroptotic pathway, which is activated by Aβ and hyperphosphorylated tau, is also implicated in AD. Mechanistically, inflammasomes, including NLRP1, AIM2 and NLRP3 inflammasome, can be activated by Aβ or hyperphosphorylated tau, ultimately leading to GSDMD‐dependent neuronal pyroptosis *in vitro* and *in vivo*.^73^, ^74^, ^75^ These studies extend our understanding of the pathogenesis of AD, which points to the modulation of pyroptosis as a novel therapeutic strategy for AD.

### 3.4 Pyroptosis in cancer

**FIGURE 5:**
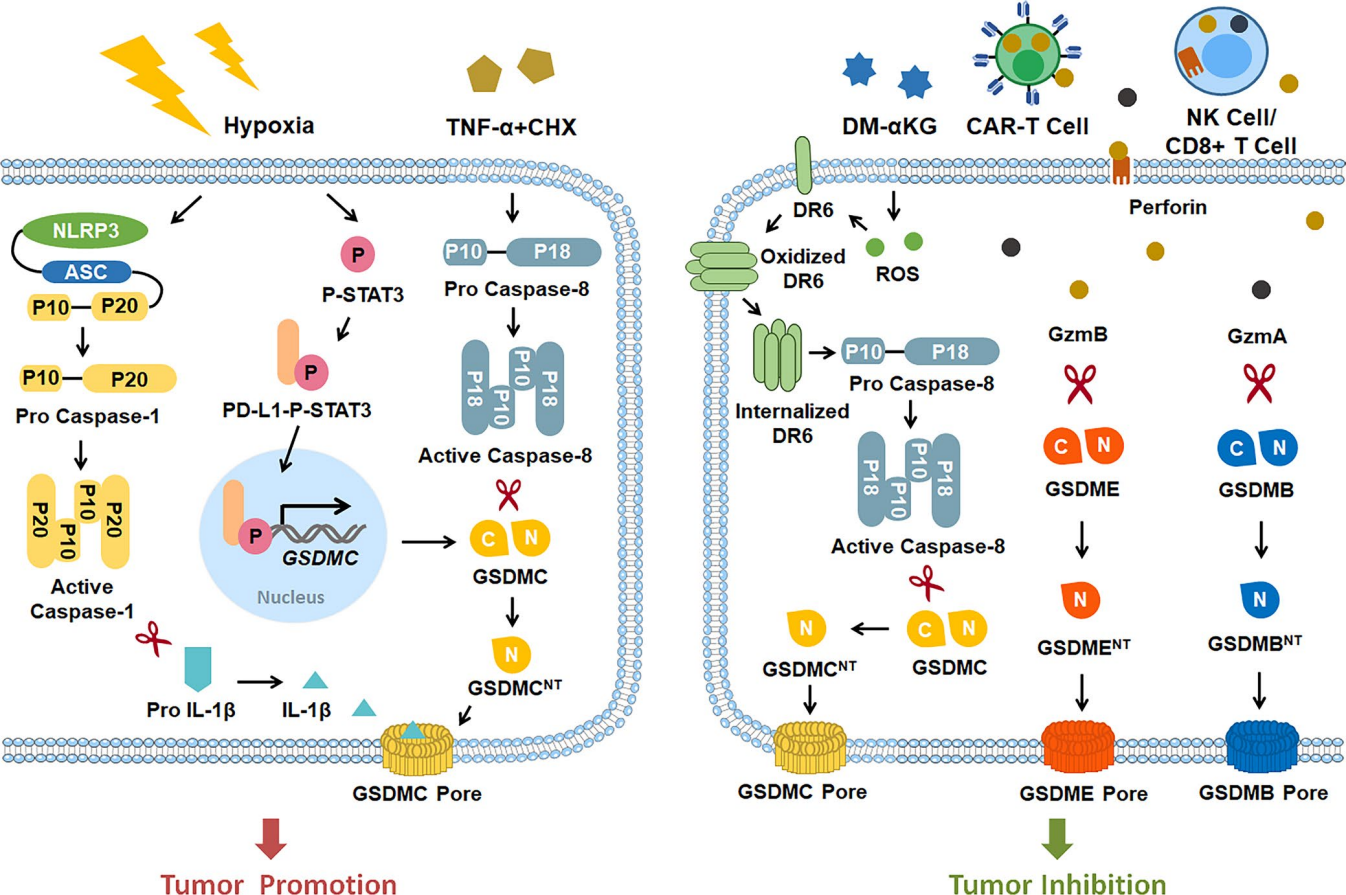
Role of pyroptosis in tumours. Pyroptosis is a double‐edged sword in tumours. On one side, pyroptosis contributes to tumour development via GSDMC‐regulated pyroptosis with hypoxia condition or TNF‐α treatment. In contrast, pyroptosis suppresses tumour promotion by DM‐α‐KG/ CAR‐T cells/ cytotoxic CD8^+^ T cells/ cytotoxic NK cells‐induced GSDMC‐, GSDMB‐ or GSDME‐mediated pyroptosis. CAR‐T cell, Chimeric Antigen Receptor T cell; CHX, cycloheximide; DM‐α‐KG, dimethyl‐α‐ketoglutarate; DR6, death receptor 6; GzmA, granzyme A; GzmB, granzyme B; NK cells, natural killer cells; PD‐L1, programmed cell death ligand 1; STAT3, signal transducer and activator of transcription

The role of pyroptosis in cancer is much more complex, which is influenced by many factors, including tissues source and genetic backgrounds (Figure 5). On the one hand, pyroptotic proteins might function as oncogenes in multiple tumours. For example, GSDMB may act as an oncogene to promote tumorigenesis in the liver, gastric tissues, uterine, cervical and breast cancers.^76^ Additionally, *GSDMB* gene amplification and protein overexpression predict the poor clinical outcomes to HER2‐targeted therapy in HER2‐positive breast cancer.^77^, ^78^ Similarly, GSDMC, another member of gasdermin family, also plays an oncogenetic role in colorectal cancer.^79^ Recently, Hou J and his colleagues indicated that GSDMC‐mediated pyroptosis in cancer cells, which is activated by PD‐L1 (programmed cell death ligand 1)‐STAT3 (signal transducer and activator of transcription)‐caspase‐8 signalling pathway under hypoxia, exacerbates chronic inflammatory necrosis in the nearby tumour microenvironment and subsequently promotes tumour angiogenesis, eventually fuelling tumour progression *in vivo*.^32^ Moreover, pyroptosis can also promote tumour cell growth via releasing inflammatory cytokines. Huang et al^80^ documented that activation of NLRP3 inflammasome contributes to carcinogenesis in head and neck squamous cell carcinoma via promoting chronic inflammation or angiogenesis.

On the other hand, several lines of evidence implicated that pyroptotic proteins may serve as tumour suppressors. Sasaki et al showed that GSDMA, which is downstream of transforming growth factor‐β (TGF‐β), is highly expressed in the gastric epithelial cell lines but appears silenced in gastric cancer cell lines.^81^, ^82^ Similarly, GSDME, which is also highly expressed in normal tissue, is downregulated by promoter DNA methylation in colorectal cancer and breast cancer.^83^, ^84^ Since GSDMA and GSDME exert critical tumour‐suppressive effects on tumorigenesis, upregulation of GSDMA/GSDME and induction of GSDMA/GSDME‐related pyroptosis might be a promising therapeutic target for the treatment of cancer. Chemotherapeutics, such as doxorubicin, lobaplatin, cisplatin and tetraarsenic hexoxide, can suppress the growth of neuroblastoma, melanoma, colon cancer cells and breast cancer by inducing GSDME‐mediated pyroptosis.^85^, ^86^, ^87^, ^88^ Recently, Zhang et al^33^ showed that intratumoral injection of DM‐αKG can notably repress tumour growth and metastasis through caspase‐8‐GSDMC‐mediated pyroptosis. Moreover, GSDME/GSDMB‐dependent pyroptosis is implicated in cytotoxic T lymphocytes or chimeric antigen receptor (CAR) T cell‐mediated antitumour immunity. For example, Zhang et al^36^ indicated that GzmB, which is released from natural‐killer (NK) and CD8^+^ T lymphocytes, can directly cleave GSDME and consequently enhance the anti‐tumour immunity by activating GSDME‐mediated pyroptosis in breast cancer cells and melanoma. Analogously, Zhou et al^37^ showed that GzmA, which is also derived from cytotoxic T cells and NK cells, can enhance tumour clearance via directly triggering GSDMB‐mediated cancer cell pyroptosis. Additionally, CAR T cell‐released GzmB triggers GSDME‐mediated pyroptosis in target tumour cells.^89^ Interestingly, Wang and colleagues further illustrated that pyroptosis can also augment antitumour immunity by sensitizing 4T1 tumours to anti‐PD1 therapy in a bioorthogonal system.^90^ Collectively, these results indicate that pyroptosis and pyroptotic proteins can exert tumour suppressive effects via induction of pyroptotic cancer cell death and enhancement of anti‐tumour immunity.

Taken together, these studies provide a comprehensive view of pyroptosis in cancer. The specific role and mechanism of pyroptosis in tumorigenesis warrant further investigations.

### 3.5 Pyroptosis in Atherosclerosis

Atherosclerosis (AS) is characterized by thickening and narrowing of the artery that occurs with the formation of atherosclerotic plaques within the arterial intima. Damage of vascular endothelial cells (VECs), infiltration of monocyte/macrophage and migration of vascular smooth muscle cells (VSMCs) in the vascular intima layer are implicated in the pathogenesis of AS.^91^, ^92^, ^93^ Accumulated evidence indicates that pyroptotic cell deaths of VECs, monocyte/macrophage and VSMCs as well as pyroptotic inflammatory responses contribute to AS.^94^

VECs pyroptosis, which is activated by oxidized low‐density lipoprotein (oxLDL)/lysophosphatidylcholine/cadmium/nicotine/ low shear stress/ trimethylamine N‐oxide (TMAO), promotes AS development by inducing the loss of endothelium integrity and increasing vascular permeability. Mechanistically, oxLDL triggers VECs pyroptosis via upregulation of TREM‐168 or through miR‐125a‐5p/Tet methylcytosine dioxygenase 2 (TET2) pathways.^95^ Lysophosphatidylcholine, a major lipid component of the plasma membrane, induces NLRP3 inflammasome‐mediated GSDMD‐dependent pyroptosis in VECs.^96^ Cadmium activates the NLRP3 inflammasome in VECs by ROS, ultimately resulting in endothelial pyroptosis.^97^ Nicotine, a major preventable risk factor for atherosclerosis, can lead to VEC pyroptosis and subsequently secretes inflammatory cytokines by ROS/NLRP3/caspase‐1 pathway.^98^ Low shear stress, which is a critical contributor to AS, induces mitochondrial dysfunction and subsequently triggers VEC pyroptosis by reducing the expression of Ten‐Eleven Translocation 2 methylcytosine dioxygenase and enhances the expression of mitochondrial respiratory complex II SDHB (subunit succinate dehydrogenase B)/ROS.^99^ Additionally, TMAO can also promote VEC pyroptosis via SDHB/ROS pathway similar to low shear stress.^100^

In addition, the death of macrophages caused by pyroptosis in AS lesions results in enhancing the inflammatory responses and synthesizing matrix metalloproteinases, which in turn advances AS lesions.^101^ Mechanistic studies showed that oxLDL, one of the key risk factors for AS, mediate macrophage pyroptosis through the TLRs/NF‐κB/NLRP3 inflammasome pathway.^102^ Additionally, oxLDL can also induce macrophage pyroptosis through ROS/NLRP3 inflammasome pathway.^103^ Nicotine, another critical risk factor for AS, triggers VECs injury and consequently exacerbates AS via a TXNIP (Thioredoxin Interacting Protein)/NLRP3/caspase‐1/GSDMD‐ mediated pyroptotic pathway in macrophages.^98^ Recently, Fidler et al showed that AIM‐caspase‐1‐ GSDMD‐mediated pyroptosis in macrophage is critical for AS in clonal haematopoiesis. Given *JAK2* ^V617F^ (*JAK2* ^VF^) mutation is one of the major risk somatic mutations in clonal haematopoiesis, the researchers used *JAK2* ^VF^ mice to explore the underlying mechanisms by which *JAK2* ^VF^ mutation gives rise to AS in clonal haematopoiesis. They found that *Jak2* ^VF^ macrophages display increased oxidative DNA damage and thereby leads to AIM2 inflammasome activation, ultimately aggravating atherogenesis by promoting caspase‐1‐GSDMD‐dependent pyroptosis in macrophage.^104^

Even VSMCs exert protective effects on the early phase of AS, however, the pyroptotic death of VSMCs contributes to AS in the later stage. Pyroptosis in VSMCs results in exacerbating the inflammatory responses and rendering plaque fragile and unstable. Pathologically, AIM2 inflammasome, which is upregulated by oxLDL, mediates GSDMD‐dependent VSMCs pyroptosis through ASC/caspase1 pathway.^105^ Additionally, NLRP3 inflammasome can be activated by oxLDL, finally resulting in VSMCs pyroptosis and progressing the pathological condition of AS.^106^ Recently, Liu et al^107^ demonstrated that dysfunctional noncoding RNAs contributes to AS by inducing VSMCs pyroptosis. Mechanistically, circular RNA PPP1CC triggers *Porphyromonas gingivalis*‐LPS‐mediated VSMCs pyroptosis by competitively sponging miR‐103a‐3p/miR‐107 and consequently resulting in HMGB1 (high‐mobility group box 1)/TLR9 (toll‐like receptor 9)/AIM2 pathway activation.

Together, these findings identify pyroptotic deaths of VECs/macrophages/ VSMCs as well as pyroptotic inflammatory responses are critical contributors to AS. Targeting the pyroptotic signalling pathway does open a new therapeutic avenue for AS.

### 3.6 Pyroptosis in acute injury

An acute injury is an injury that usually results from a specific impact or trauma in the brain, lung or kidney. Inflammation and pyroptotic cell death, which are triggered via cytoplasmic inflammasome complexes, are regarded as key contributors to acute injuries.

Traumatic brain injury (TBI) is sudden traumatic damage in the brain with oedema, axonal shearing, neuronal death and vascular damage. The post‐TBI primary insult typically leads to secondary damage, including neuroinflammation,^108^ neuronal cell death^109^ and mitochondrial dysfunction.^110^ Growing research has revealed that neuroinflammation and neuronal pyroptotic cell death mediated by active caspase‐1 downstream of NLRP1/NLRP3/AIM2 inflammasome activation is pivotal mechanisms of brain injury responses in TBI.^111^, ^112^, ^113^ Additionally, pyroptosis of infiltrating CD11^+^ leukocytes and activated microglia contributes to the pathophysiology of secondary injury after severe TBI.^114^ Furthermore, canonical inflammasome‐induced pyroptosis in brain microvascular endothelial cells (BMVECs) results in blood‐brain barrier (BBB) leakage and brain oedema, ultimately aggravating damages after TBI.^115^ Collectively, these results advance our understanding of pyroptosis in TBI.

Acute lung injury (ALI), which is characterized by acute severe hypoxia, is lung inflammation and VECs damage arising from a wide variety of both pulmonary and generalized acute diseases. Pathological studies indicate that alveolar macrophage activation and VECs injury are involved in the clinical features of acute lung injury. However, the detailed cellular mechanism and the potential role of alveolar macrophage activation and VECs damage in ALI remain unclear. Recently, some researches revealed that pyroptosis of alveolar macrophage and VECs contributes to the pathogenesis of ALI. At first, Kovarova et al^116^ indicated that NLRP1‐dependent pyroptosis in alveolar macrophage, which is triggered by anthrax toxin, leads to ALI. Then, three independent groups showed that alveolar macrophage pyroptosis mediated by NLRP3 inflammasome induce ALI following the cardiopulmonary bypass, bleomycin or acute respiratory distress syndrome (ARDS).^117^, ^118^ Additionally, VECs pyroptosis induced by NLRP3/ASC/caspase‐1 complex also exacerbates the pathological process of ALI.^119^, ^120^ In conclusion, these studies suggest that pyroptosis in alveolar macrophage and VECs underlies ALI, suggesting several potential therapeutic targets for ALI.

Acute kidney injury (AKI) is sudden damage of the kidney in response to many risk factors, including ischemia‐reperfusion (I/R), chemotherapy and contrast agents. Renal tubule epithelial cell death is a common and critical pathophysiological component of AKI. Recently, renal tubular epithelial cell (TECs) pyroptosis has been characterized in acute kidney injury. Yang and coworkers gave the first evidence that I/R can activate the CHOP (C/EBP homologous protein)/caspase‐11 pathway, ultimately inducing renal tubule pyroptosis in AKI.^121^ Additionally, Zhang et al^122^ gave another evidence that caspase‐11‐induced pyroptosis in TECs underlies contrast‐induced AKI. In 2019–2020, Miao et al and Li et al^123^, ^124^ simultaneously demonstrated that caspase‐11 cleaves GSDMD to induce GSDMD‐mediated pyroptosis and urinary inflammatory cytokine excretion in I/R‐ or cisplatin‐triggered AKI. Xiao et al^125^ showed that transcription factor Tisp40 (transcript induced in spermiogenesis 40), which is unregulated in TECs after renal I/R injury, induces GSDMD‐dependent pyroptosis in I/R‐triggered AKI by activating NF‐κB signalling. Of interest, Xia et al^126^ further illustrated that GSMDE, which is another critical executor of pyroptosis, is also involved in cisplatin‐ or I/R‐induced AKI. Mechanistically, active caspase‐3, which is activated after I/R‐ or cisplatin‐treatment, cleaves GSDME and consequently contributes to I/R‐ or cisplatin‐induced AKI by triggering GSDME‐mediated pyroptosis in TECs. Together, all of these new findings confirmed that pyroptosis of TECs plays a critical role in AKI, indicating the potential for developing novel treatment against AKI by targeting the pyroptotic signalling pathway.

### 3.7 Pyroptosis in adverse pregnancy complications

Pregnancy complications, such as preeclampsia (PE), gestational diabetes and preterm birth, are health problems that occur during pregnancy. Adverse pregnancy complications may have serious effects on the pregnant woman and her baby. Therefore, clinical doctors and scientists try to protect pregnant woman from adverse pregnancy complications by figuring out the underlying cellular mechanisms in the pathogenesis of adverse pregnancy complications.

PE, a pregnancy‐specific hypertensive syndrome characterized by the onset of hypertension and proteinuria after 20 weeks of gestation, is a severe complication of pregnancy affecting more than 3–5% of pregnancies worldwide.^127^ Pathological studies indicate that placental inflammation is implicated in the aetiology of PE. However, the detailed cellular mechanism of placental inflammation in PE remains unclear. In 2019, Cheng and coworkers gave their first evidence that pyroptosis in the placenta promotes early‐onset PE. Mechanistically, NLRP3 inflammasome, which is activated by endoplasmic reticulum (ER) stress/unfolded protein response (UPR)/thioredoxin‐interacting protein (TXNIP) pathway upon hypoxia, triggers GSDMD‐mediated pyroptotic cell death and sterile inflammation in human trophoblasts and eventually results in early‐onset PE.^128^ Additionally, Liu et al^129^ gave another evidence that pyroptosis plays a pivotal role in PE by promoting angiotensin II type 1 receptor autoantibody (AT1‐AA) production. Mechanistically, they showed that caspase‐1, a key component of the pyroptotic signalling pathway, is upregulated and hyperactivated in PE. Hyperactivated caspase‐1 enhances AT1‐AA production and trophoblast pyroptosis, ultimately leading to PE. Recently, Quan et al^130^ demonstrated that chemerin‐induced trophoblasts pyroptosis aggravates PE through CMKLR1 (chemokine‐like receptor 1)/AMPK (activated AMP‐activated protein kinase)/TXNIP (thioredoxin‐interacting protein)/NLRP3 pathway upon hypoxia/reoxygenation (H/R). Collectively, these studies suggest that placental pyroptosis is a key pathogenic event in PE. Therefore, targeting pyroptosis might be a promising therapeutic way for the treatment of PE.

Preterm birth, which is defined as a baby birth before spontaneous preterm labour, is a leading cause of perinatal morbidity worldwide. Although there are lots of putative causes of preterm labour, only intra‐amniotic inflammation has a clear causal relationship with preterm birth. Of note, several recent studies demonstrated that pyroptosis‐induced intra‐amniotic inflammation plays a pivotal role in preterm birth. In 2015, Whidbey et al^131^ first revealed that Group B streptococci (GBS), which is a critical pathogen to trigger intra‐amniotic inflammation, can induce preterm birth through a combined action of macrophages pyroptosis and red blood cells (RBC) lysis. Mechanistically, GBS pigment can cause macrophages pyroptosis via the NLRP3‐caspase‐1 pathway in culture THP‐1 macrophages. In addition, GBS lipid toxin/pigment can also directly penetrate the RBC membrane and subsequently induces RBC lysis through a colloidal osmotic mechanism *in vitro*. During 2017–2019, Gomez‐Lopez et al further showed that the expression of NLRP3, GSDMD, IL‐1β and IL‐18 are upregulated in chorioamniotic membranes from women in spontaneous preterm labour with acute intra‐amniotic inflammation/infection.^132^, ^133^ In addition, the level of GSDMD is also increased in amniotic fluid from women with intra‐amniotic inflammation/infection.^132^ Moreover, Gomez‐Lopez et al^132^ indicated that the leukocytes and decidual stromal cells, which were isolated from women with preterm labour and birth, undergo caspase‐1‐mediated pyroptosis. Together, these studies identified that pyroptosis is implicated in the pathogenesis of preterm birth, which may provide a new therapeutic target for preventing preterm birth in future.

### 3.8 Pyroptosis as therapeutic targets

Given that pyroptosis takes a prominent role in these noninfective diseases, the development of small molecular inhibitors targeting pyroptotic proteins and signalling pathway is a promising therapeutic strategy (Table 1).

**TABLE 1:** Therapeutic agent for pyroptotic signalling pathway

Targets	Agents	Diseases	Outcome of inhibition	Ref.
NLRP3	MCC950	Stroke	MCC950 provides protection in mouse model of tMCAO	136
		AD	MCC950 promotes non‐ phlogistic clearance of amyloid‐β and improves cognitive function in APP/PS1 mice	137
		Epilepsy	MCC950 suppresses NLRP3 ‐mediated inflammation in the hippocampus of SE mice model	138
		PD	MCC950 attenuates dopaminergic neuronal degeneration, neuroinflammation and behavioural deficits in a MPTP‐induced PD mice model	139
		AS	MCC950 inhibits atherosclerotic lesion development	140
		SCCHN	MCC950 delays tumorigenesis in Tgfbr1/ Pten 2cKO mouse SCCHN model	80
	Ghrelin	MS	Ghrelin relieves demyelination and neuroinflammation in MS	141
	Salidroside	PD	Salidroside ameliorates PD	142
		AS	Salidroside decreases the formation of atherosclerosis plaque through suppressing NLRP3‐related VECs pyroptosis	143
NLRP1	SDG	IBD	SDG relieves colitis by inhibiting NLRP1 inflammasome	144
AIM2	A151	Stroke	A151 prevents microglial pyroptosis, finally reducing inflammation‐diminishing cell death and attenuating infarct volume	65
Caspase‐1	Vx765	Stroke	Vx765 reduces neuronal death in a murine model of cerebral ischemia	146
		AD	Pre‐symptomatic treatment with VX‐765 delays glial activation and spatial memory deficits in APP^Sw/Ind^ mutant J20 mice	147
		AS	VX‐765 attenuates AS in ApoE deficient mice	106
		TBI	VX765 counteracts neurological damage after TBI by reducing caspase‐1‐mediated pyroptosis	148
		MS	VX‐765 prevents demyelination, axonal injury and improved neuro‐behavioural performance in MS via inhibition of caspase‐1	149, 150
	LXA_4_	PE	LXA_4_ attenuates AT1‐AA production and trophoblasts pyroptosis in PE through suppressing caspase‐1 activity	129
Caspase‐1/4/5/11	Ac‐FLTD‐CMK	TBI	Ac‐FLTD‐CMK suppresses neuronal death and inflammation after TBI	152
Capsase‐3	Ac‐DMPD/ DMLD‐CMK	Acute hepatic failure	Ac‐DMPD/DMLD‐CMK alleviates hepatocyte injury in a mouse model of acute hepatic failure	153
GSDMB	Anti‐GSDMB antibody	Cancer	Anti‐GSDMB antibody reduces tumour growth and lung metastasis	159
GSDMD	NSA	AD	NSA pre‐treatment inhibits Aβ_1‐42_‐induced pyroptosis in cortical neurons *in vitro*	73
	Disulfiram	MS	Disulfiram relieves demyelination and neuroinflammation in MS	156
	DMF	MS	DMF reduces neuropathology and demyelination in MS	158
		FMF	DMF alleviates GSDMD‐ dependent pyroptosis and tissue damage *MefvV* ^726A/V726A^ FMF mouse model	158
GSDME	2‐BP	Cancer	2‐BP counteracts chemotherapy drugs‐induced GSDME‐ mediated pyroptosis	87
	DMF	MS	DMF reduces neuropathology and demyelination in MS	158
		FMF	DMF alleviates GSDMD‐induced injury in *Mefv* ^V726A/V726A^ FMF mouse model	158

Abbreviations: AT1‐AA, angiotensin II type 1 receptor autoantibody; Ac‐FLTD‐CMK, *N*‐acetyl‐Phe‐Leu‐Thr‐Asp‐chloromethylketone; AD, Alzheimer's disease; AS, atherosclerosis; AKI, acute kidney injury; 2‐BP, 2‐bromopalmitate; DMF, dimethyl fumarate; FMF, familial Mediterranean fever; IBD, inflammatory bowel disease; LXA_4_, Lipoxin A_4_; MS, multiple sclerosis; NSA, necrosulfonamide; PD, Parkinson's disease; PE, preeclampsia; SCCHN, squamous cell carcinoma of the head and neck; SDG, secoisolariciresinol diglucoside; SE, status epilepticus; TBI, traumatic brain injury; tMCAO, transient middle cerebral artery occlusion; VECs, vascular endothelial cell; VSMCs, vascular smooth muscle cells.

The activation of inflammasomes is the starting point of pyroptosis. Therefore, it is the star member of the inflammasomes family, targeting NLRP3 attracts the most attention. MCC950, which is a potent, selective and small‐molecule inhibitor of NLRP3,^134^, ^135^ can relieve the pathological progression of various noninfective diseases, including ischemic stroke,^136^ AD,^137^ epilepsy,^138^ PD,^139^ AS^140^ and squamous cell carcinoma of the head and neck (SCCHN).^80^ Additionally, Ghrelin and Salidroside, which can also inhibit the activity of NLRP3 inflammasome, have recently been proved to ameliorate MS, PD and AS through suppressing NLRP3‐dependent pyroptosis.^141^, ^142^, ^143^ Other inflammasomes, such as NLRP1 and AIM2, also draw researchers’ attention. Secoisolariciresinol diglucoside (SDG), a plant lignan isolated from flaxseed, suppresses colitis by inhibiting the activation of NLRP1 inflammasome.^144^ The cytosolic dipeptidyl peptidases 8 and 9 (DPP8/DPP9) have been reported to suppress NLRP1 inflammasome activity by directly binding to and sequestering NLRP1 inflammatory C‐terminal fragment (NLRP1 CT).^145^ It will be interesting to test the therapeutic effects of DPP8/DPP9 on the NLRP1‐mediate pyroptosis‐related diseases in the future. In addition, A151, an antagonist of AIM2 inflammasome, prevents microglial pyroptosis and finally ameliorates brain injury after ischemic stroke.^65^

Caspase is the key link in the process of pyroptosis. Active caspases can cleave gasdermins and pro‐inflammatory cytokines, finally driving pyroptosis. Therefore, targeting caspases is an attractive strategy for the inhibition of pyroptosis. For example, caspase‐1 inhibitor Vx765, also named Belnacasan, can attenuate pyroptosis to ameliorate the damage after stroke,^146^ AD,^147^ AS,^106^ TBI^148^ and MS.^149^, ^150^ Additionally, Lipoxin A_4_ (LXA_4_), an endogenous dual anti‐inflammatory and pro‐resolving lipid mediator can prevent PE through inhibiting caspase‐1 activation.^129^ Furthermore, *N*‐acetyl‐Phe‐Leu‐Thr‐Asp‐chloromethylketone (Ac‐FLTD‐CMK), a GSDMD‐derived inhibitor, specifically suppresses inflammatory caspases (caspase‐1/4/5/11) through extensively hydrophilic and hydrophobic enzyme–inhibitor interactions.^151^ Recently, Wang et al^152^ showed that Ac‐FLTD‐CMK exerts neuroprotective effects on TBI through inhibiting inflammatory caspases‐dependent pyroptosis. Ac‐DMPD and DMLD‐CMK, which are newly identified GSDME‐derived, specific caspase‐3 inhibitors, protect hepatocytes and macrophages against bile acid‐induced pyroptosis and apoptosis *in vitro* and *in vivo*.^153^

Since gasdermins family is the executor protein in pyroptosis, the identification of drugs targeting gasdermin proteins engages researchers’ attention. Necrosulfonamide (NSA), which is identified to inhibit the formation of GSDMD^NT^ fragment by binding to C191 amino acid,^154^ suppresses Aβ_1‐42_‐induced pyroptosis *in vivo*.^73^ Similar to NSA, disulfiram, which also potently inhibits GSDMD‐induced pyroptosis through regulation of C191 amino acid,^155^ relieves MOG_35‐55_ peptide‐triggered demyelination and neuroinflammation in MS.^156^ Punicalagin, an antioxidant polyphenol from pomegranates, might prevent pyroptosis and subsequent IL‐1β secretion through inhibition of GSDMD^NT^ insertion into the plasma membrane.^157^ Recently, Humphries et al^158^ showed that dimethyl fumarate (DMF), which can inactivate GSDMD and GSDME by succinating the GSDMD at Cys^191^ and GSDME at Cys,^45^ respectively, reduces neuropathology and demyelination in MS. DMF can also alleviate GSDMD‐derived pyroptosis in *Mefv* ^V726A/V726A^ familial Mediterranean fever (FMF) mouse model.^158^ Additionally, 2‐bromopalmitate (2‐BP), which could inhibit the palmitoylation of C‐terminal domain GSDME and increase the interaction between C‐ and N‐ terminal domain of GSDME, counteracts chemotherapy drugs‐induced GSDME‐mediated pyroptosis in the cancer cells.^87^ Furthermore, Molina‐Crespo et al^159^ developed nano‐sized anti‐GSDMB antibodies to suppress the migration and metastasis of HER2 breast cancer *in vitro* and *in vivo*.

## 4 CONCLUSIONS AND FUTURE PERSPECTIVES

Pyroptosis, a kind of inflammatory cell programmed death mediated by gasdermins protein, is an important part of innate immunity. Moreover, pyroptosis also exerts a vital role in noninfective inflammatory disorders. Our cognition of pyroptosis has gone through several stages. From the beginning, researchers just focused on the essential role of canonical or noncanonical inflammasomes (such as NLRP1, NLRP3, NLRC4 and AIM2 inflammasome) and pro‐inflammatory caspases (caspases‐1/4/5/11) in pyroptosis. Subsequently, researchers discovered that pro‐apoptosis caspases (caspases‐3/6/8) also participate in the process of pyroptosis. Until recently, several researches illustrated that granzymes (GzmA/GzmB) can initiate pyroptosis without caspases participation. These studies refresh our understanding of pyroptosis. Further studies will be continued to explore the precise activation modes of pyroptosis in the future.

Growing evidence indicates that pytoptosis is implicated in multiple noninfective diseases, such as sterile inflammatory diseases, autoimmune diseases, neuronal diseases, atherosclerosis, acute injuries and various cancers, thereby providing a new entry point for the treatment of these disorders. However, some inhibitors, such as caspase antagonists, NSA and disulfiram, might lead to unexpected side effects due to the lack of sufficient specificity. Further researches are needed to improve the specificity of pyroptotic inhibitors.

Although pytoptosis exerts pathogenic effects on noninfective diseases, it also has beneficial effects on tumour suppression in some contexts. For instance, Wang and his colleagues demonstrated that a small amount of pyroptotic tumour cell death (less than 15%) is sufficient to clear the entire tumour graft.^90^ Additionally, several studies demonstrated that GSDMB or GSDME‐mediated pyroptosis, which is triggered by granzymes released from cytotoxic lymphocytes, can potently suppress tumour growth.^36^, ^37^ These studies suggest that enhancing pyroptosis does open novel therapeutic avenues for cancer clearance via increasing pyroptotic cell death and anti‐tumour immunity. However, pyroptosis might be a double‐edged sword. Extensive pyroptosis can cause severe tissues damages. Shen et al^160^ indicated that GSDME‐mediated pyroptosis in renal TECs is responsible for cisplatin‐ or doxorubicin‐induced nephrotoxicity. Moreover, GSDME‐dependent pyroptosis and subsequent IL‐1β/IL‐6 releases in macrophages contribute to cytokine release syndrome (CRS) during CAR T cell therapy.^89^ Thus, specific activation of a pyroptotic signalling pathway in cancer cells rather than normal cells is critical for pyroptosis‐related tumour therapy. Future studies are warranted to develop tumour‐specific pyroptotic treatment.

Recently, Hansen et al showed that enteroinvasive *Shigella flexneri* (*S*. *flexneri)* can resist NK cell‐induced bactericidal functions by counteracting NK cell‐activated GzmA/GSDMB pyroptotic signalling pathway. Mechanistically, IpaH7.8, which is the *S*. *flexneri* type 3 secretion system (T3SS) effector protein, selectively binds and ubiquitinates GSDMB, eventually inducing GSDMB degradation and preventing NK cell‐GzmA‐GSDMB‐mediated pyroptotic lysis of *S*. *flexneri*.^161^ This study suggests that post‐translational modification, such as ubiquitination, might serve as an important way to enhance or antagonize pyroptosis. It will be very attractive to figure out the potential regulatory effects of other post‐translational modifications on pyroptosis in the future.

In conclusion, pyroptosis, which is an important kind of inflammatory program cell death, plays a key role in noninfective inflammatory disorders. Future studies are needed to further demonstrate its definite role in human diseases, providing a unique therapeutic opportunity for the treatment of multiple sterile inflammatory disorders.

## CONFLICT OF INTEREST

The authors have declared no conflicting interests.

## AUTHOR CONTRIBUTION

T.L. and L.T. wrote the manuscript; G.Z., B.L. and L.T. edited the paper.

## Data Availability

The data that support the findings of this study are available from the corresponding author upon reasonable request.
